# 

**Published:** 1867-06

**Authors:** 


### BOOK NOTICES.

*Treatment of Fractures of the Lower Extremities by the use of the Anterior Suspensory Apparatus.*—By N. R. Smith M. D., Professor of Surgery in the University of Maryland. Baltimore: Kelly & Piet, 1867.

No more valuable improvement in the Surgical treatment of fractures than Professor Smith's Anterior Splint has been made during this century. It has passed so generally into common use, that the rising generation of surgeons find it difficult to estimate the magnitude of this boon to suffering humanity. Those, however, who remember the torture of the old fracture-box and the straight splint, have better facilities for determining the value of this great improvement in the treatment of fractures of the thigh. They have seen a patient lying for weeks on his back, with a long straight splint reaching from his arm-pit to a point considerably below the sole of his foot, another on the inside stretching down from the perineum, with the foot strapped down to one and copious turns of bandage holding both firmly to the broken limb. The poor sufferer was a picture of immoveable wretchedness, his whole frame fixed as in some mediæval instrument of torture, and while he waited in rigid misery for the bones to knit, the constant pressure as he lay on his back gave him bed sores which, owing to the unchangeable position, it was impossible alike to avoid or successfully to treat. In addition to other troubles, the unspeakable weariness of the fixed attitude wore out the patient, and the weak sufferer died less of the accident than of the treatment.

With all this trouble Dr. Smith's apparatus enables us to dispense. The principles of treatment which have always been recognized are met in it by arrangements peculiarly comfortable to the patient, and new principles have been introduced which have added largely to the ease of the sufferer. Every one saw that it was necessary to bring the ends of the broken bone in apposition and to keep them there. It was well known that the main difficulty was the tendency of the trunk to sink down in the bed and thus to push the upper fragment past the lower and to shorten the limb. To meet the indications of treatment, the best mechanical contrivance was a long, straight immoveable splint which should serve as a point of attachment wherefrom to hold up the upper fragment and to draw down the lower.

Dr. Smith, however, conceived the happy idea of giving the patient a chance to change his position. His apparatus is too well known to every one who has studied surgery in Baltimore, to need a description for this latitude, but as we have readers at a distance to whom its details are not familiar, we shall give them a brief outline of its construction and application. Its immovable portion consists of a single splint, with one angle at the ankle, another at the knee, and a third at the hip. It is made sometimes of wood, but preferably of wire. It must be long enough to stretch from the anterior spinous process of the ilium to an inch beyond the toes when the whole limb is extended. With the wire splint, the angles are easily modified to suit particular fractures. Another essential part of the apparatus is the suspensory band. This consists of a cord attached by either end to the splint, and supporting it from the thigh and the shin, and fixed by another cord to a pulley on the ceiling. The anterior splint is laid upon the limb, and made fast at the foot, the ankle, above and below the knee, and near the hip. The cord is then attached and the limb hoisted by means of the pulley. It is now easy to envelope limb and splint with a roller, which affords a support at once firm and easy. The patient lies comfortably in bed, shifting his position at will, without fear of disturbing the fragments; for extension and counter-extension being made by splint and cord, by regulating the direction of the suspension, no undue traction is made upon the limb, and no severe local pressure exists at any one portion of the limb. It is the most comfortable fracture apparatus in existence, and no surgeon who has once applied it will ever use any other. It is applicable to all fractures of the thigh and leg.

The book before us is a full and satisfactory description of this invaluable apparatus and the proper method of applying it. It is illustrated by a sufficient number of wood-cuts for a clear understanding of the subject. It contains furthermore reports of several cases in which the application of the splint and its results are described. The most striking of these is the summary of cases of compound comminuted fractures of the thigh produced by gunshot wounds, treated in the Confederate hospitals at Richmond, during the second year of the war. From this summary it appears that death inevitably followed every attempt to save the limb, except in those cases in which this suspensory apparatus was applied. Of these one half recovered. This simple statement speaks volumes for Professor Smith's method of treatment.

The publishers deserve much credit for the handsome style in which they have gotten up the book. It is elegantly printed on fine tinted paper and neatly bound in cloth.

### EDITORIAL DEPARTMENT.

*Iowa State Dental Society.*—Dr. H. S. Chase, the Corresponding Secretary of this Society, wishes us to announce that the next session will be held at Lyons, on Tuesday, July 9th, at 7½ o'clock, P. M. There will be a session of two whole days. Besides the appointed essayists, several distinguished Dentists are expected from abroad. From the reputation of the essayists and the subjects of the essays, this meeting will no doubt be an interesting one.

The members are requested to bring with them microscopical specimens of teeth; dental curiosities; new or improved instruments; casts of malformations, &c. Also, microscopes of 300 diameters and over.

---

*Massachusetts Dental Society.*—The Annual Meeting of this Society was held on the 23d of May at their Hall in Boston. The Annual address was delivered by Dr. Henry F. Bishop of Worcester. Clinics were held by Drs. E. N. Harris, G. T. Moffatt, S. J. McDougall, and T. B. Hitchcock.

---

*Dr. Noel's Review of Dr. Arthur's Book on Decay of the Teeth.*—We are sorry to announce to our readers, that owing to the great pressure on our columns, this article has been unavoidably crowded out. It shall certainly appear in our next.

